# Smoking cessation advice and quit attempts in South Africa between 2007 and 2017: A cross-sectional study

**DOI:** 10.18332/tid/132148

**Published:** 2021-02-11

**Authors:** Olalekan A. Ayo-Yusuf, Olufemi B. Omole

**Affiliations:** 1 Africa Centre for Tobacco Industry Monitoring and Policy Research, Sefako Makgatho Health Sciences University, Pretoria, South Africa; 2 Division of Family Medicine, Faculty of Health Sciences, University of the Witwatersrand, Johannesburg, South Africa

**Keywords:** quit advice, smoking, quit attempt, FCTC Article 14, e-cigarette

## Abstract

**INTRODUCTION:**

The aim of this study was to determine trends in receiving quit advice from healthcare professionals among current smokers in South Africa, pre- and post-adoption of Article 14 guidelines of the WHO FCTC in 2010, and to determine the association between quit advice, e-cigarette use and quit attempt.

**METHODS:**

This study was a secondary data analysis involving 2206 ever-smokers aged ≥16 years who participated in the South African Social Attitude Surveys conducted in 2007, 2010 and 2017. Data included participants’ sociodemographics, tobacco, and/or e-cigarette use (for years 2010 and 2017 only), exposure to others’ smoking at home and/or work or public places, quit advice, and quit attempts. Analyses included chi-squared test and logistic regression.

**RESULTS:**

The mean cigarettes smoked per day, the proportions of smokers offered quit advice, planning to quit and who made a quit attempt did not change significantly between 2007 and 2017 (p=0.67, p=0.70, p=0.09 and p=0.40, respectively). However, there was a marginally significant increase in e-cigarette uptake between 2010 and 2017 (p=0.05). In a bivariate analysis, quit advice was significantly associated with making a quit attempt across all survey years. In the final multivariable-adjusted regression model, having received a quit advice (OR=1.967; 95% CI: 1.255–3.083) compared to not, and being Colored/mixed race (OR=0.467; 95% CI: 0.298–0.732) compared to self-identifying as Black African, remained independently associated with making a quit attempt.

**CONCLUSIONS:**

Except for marginally increased e-cigarette use, there was no significant change in smoking or quitting behavior in South Africa post-adoption of Article 14 guidelines. The study findings highlight the importance of quit advice in promoting quitting behavior and suggest the need to scale it up in South Africa.

## INTRODUCTION

Smoking rates are high in most developing countries including South Africa, and if left unchecked, will be the leading single cause of death by 2030 in most of these countries^1^. Despite the progressive tobacco control program in South Africa (mostly centered on aggressive tax and price hikes on tobacco products) and a reduction in smoking prevalence^2,3^, tobacco use still ranks among the leading causes of premature morbidity and mortality in the country^4,5^. In the last two decades in South Africa, the reported prevalence of daily smoking has decreased from 42% to 37% among men, and 11% to 8% among women^6,7^. However, smoking rates remain unacceptably high among the Colored (mixed ancestry) population, where it is reported to be 49.3% among men and 38.1% among women, in 2017. In addition, an estimated 51.2% and 10.4% of tobacco and non-tobacco users, respectively, are still regularly exposed to secondhand smoke (SHS) at home or outside of home.

In 2010, the Conference of Parties (CoP) to the WHO Framework convention on tobacco control (FCTC) adopted Article 14 guidelines to encourage all parties to implement measures to promote cessation of tobacco use by providing adequate treatment for tobacco dependence^8^. This requires healthcare professionals to, among other things, screen for tobacco use and provide brief advice and offer cessation treatments during clinical encounters. Each of these activities when performed by a healthcare professional has been shown to significantly increase the likelihood of smoking cessation^9^. However, healthcare professionals do not consistently perform the activities spelt out in the Article^10^. Indeed, only about 29.3% of smokers received quit advice from a healthcare professional in 2012 in South Africa^3^.

Although South Africa is a signatory to the WHO FCTC and is one of few African countries that have made meaningful efforts to incorporate tobacco dependence treatment into their healthcare systems^11^, there are no published data indicating whether the adoption of Article 14 in 2010 has had any effect on the rate at which healthcare professionals offer quit advice to smokers or on the rate of quit attempts by South African smokers.

In recent times, e-cigarette use has been promoted as a less harmful alternative to cigarette smoking and as a cessation aid, amidst concerns about the toxicity of some of its chemical components and the potential for renormalization of smoking^12,13^. Indeed, associations have been demonstrated between e-cigarette use and increased quit attempts in some studies conducted in the US^14,15^. Other reports, including the findings of a Cochrane review of randomized control trials have also supported the assertion that nicotine e-cigarette increases the likelihood of smoking abstinence and that the risk of severe adverse effects may not be significantly different from those of nicotine replacement treatments^16,17^.

E-cigarettes, which were introduced into the South African market about the year 2008, are also becoming popular^18^ and little, or no data, exist to inform whether the observed associations elsewhere are true for South Africa. Such data would be important in informing tobacco control policy in South Africa, especially with regard to the role of e-cigarette use in promoting quitting behavior at the population level. However, it is conceivable that e-cigarette users might be those already planning to quit and have just received quit advice from a healthcare professional to facilitate cessation. The aim of this study was to determine trends in receiving quit advice from healthcare professionals among current smokers in South Africa, pre- and post-adoption of Article 14 guidelines of the WHO FCTC in 2010, and to determine the independent association between quit advice and quit attempt.

## METHODS

### Study design and population sample

This was a cross-sectional study involving a secondary data analysis of a nationally representative sample of South Africans ever-smokers aged ≥16 years that participated in the 2007 (n=764), 2010 (n=705) and 2017 (n=734) South African Social Attitude Survey (SASAS) waves that included a tobacco use module.

The South African Social Attitudes Survey (SASAS)^19^ is a nationally representative, cross-sectional survey that has been conducted on an annual basis by the Human Sciences Research Council (HSRC) since its inception in 2003. The detailed sampling procedure, which is consistent over survey waves, has been published^20,21^ and involved a multi-stage cluster sampling technique, which was used to obtain a representative sample of South Africans by population group and geographical location. Data collection was by face-to-face interviews. Information was collected on household income/income, literacy, smoking, alcohol use, the environment, national health insurance, oral health, voting, and social cohesion. Although the primary survey was part of the Human Sciences Research Council’s (HSRC’s) annual SASAS, this secondary data analysis used only a pooled sample of ever smokers that had data on variables of interest (n=2206). The pooled dataset contained no personal identifiers.

### Measures

#### Sociodemographic characteristics

These included age, educational level completed (<High school, High school, >High school), sex (male or female), ethnicity in terms of self-identification as Black African, Colored (mixed ancestry), Indian/Asian, or White, and location of residence (urban or rural).

#### Current tobacco smokers

These were defined as respondents who reported that they were currently smoking hand-rolled or commercially manufactured cigarettes, cigars, pipes, or water pipes daily, or on some days. Participants were also asked to indicate the number of cigarettes, or equivalent, smoked per day (CPD), (subsequently coded as ‘0’=1–10, ‘1’=11–20, ‘2’=21–30, or ‘3’ for >30 cigarettes).

#### E-cigarette ever use

This was defined as a participant indicating current daily or some days use, or used in the past, in both the 2010 and 2017 surveys since no question was asked about e-cigarette use during the 2007 survey because e-cigarettes were not yet marketed in South Africa.

#### Past-month exposure to secondhand smoke (SHS)

Past-month SHS exposure at home and work or public place (e.g. restaurants or bars), was defined as indicating exposure to others’ smoking in these places at least once in the last 30 days. Considering the importance of the influence of smoking cues on motivation to quit and particularly having another household member smoking, we sub-categorized the variable as: ‘No exposure to other's smoking’; ‘Exposure only at work or public space (i.e. exposure only outside of home)’; and ‘Exposure at home (with or without exposure in other places)’.

#### Planning to quit

This was defined as any response of ‘within the next month’, ‘within the next 6 months’, or ‘sometime in the future, beyond 6 months’ to the question: ‘Are you planning to quit smoking?’.

#### Quit attempt among current smokers

This was defined as any response of ‘once’, ‘twice’, ‘three times or more’ (vs ‘never’) to the question: ‘Have you ever tried to quit smoking?’, or if participants indicated having tried to quit in the last 12 months in one wave of the surveys.

#### Receipt of quit advice from a healthcare professional

This was defined as a participant reporting receipt of advice to stop smoking from any of listed health professionals (dentist and/or medical doctor and/or nurse and/or other health professions vs none).

All questions used in this survey are as previously published^20,21^.

### Statistical analysis

Analysis was performed with SPSS version 24 (IBM SPSS Statistics). All data were weighted to account for oversampling of some population groups and for the response patterns, to yield nationally representative estimates for each survey year. Outcomes of analysis included the proportions of the sample who were current smokers, the mean CPD and the prevalence of ever e-cigarette use. Furthermore, the proportions of the sample that reported exposure to SHS at home, work or in public places, received quit advice from a healthcare professional, ever made a quit attempt or planned to quit were determined. Smokers were further stratified by sociodemographic characteristics. The associations between the quitting behaviors (quit attempt, quit plan and having received quit advice) and sociodemographic and other behavioral variables were determined in bivariate analysis using chi-squared statistics to test for trends and group differences for categorical variables, and t-tests or ANOVA for continuous variables. Following the bivariate analysis, in order to determine the factors independently associated with quit attempts, multivariable-adjusted stepwise logistic regression analyses were performed, adjusting for factors associated with quit attempt in a bivariate analysis at the 10% level (p<0.10) for any survey year. However, survey year was included in all models irrespective of the level of significance since we used the pooled data from 2010 and 2017 SASAS for the regression model due to the relatively small sample of e-cigarette users for each survey year. Including survey year in the models allowed for control for potential differences in the distribution of confounders across the two survey waves, including those potential confounders that might not have been observed. The pooled data were used for the regression model in order to increase the sample size, improve the precision of parameter estimates and therefore obtain a more reliable estimation. The level of significance was set at p<0.05.

## RESULTS

Data of interest were obtained from 2206 ever smokers pooled from the three survey waves. As shown in Table 1, most participants (ever smokers) were: male (63.6%), Black African (44.8%), had less than high school education (59.5%), lived in an urban area (77.1%), currently smoke (88.0%), and were exposed to other’s smoking at home (72.2%), with or without additional exposure at work or in public places in the past one month.

**Table 1:** Study participants’ sociodemographic characteristics of the South African Social Attitude Survey (SASAS) waves of 2007, 2010 and 2017 that included a tobacco module (N=2206)*

*Characteristics*	*n*	*%*
**Age** (years)		
16–24	355	16.1
25–34	493	22.4
35–44	478	21.7
45–54	364	16.5
≥ 55	516	23.4
**Sex**		
Male	1402	63.6
Female	804	36.4
**Education level**		
<High school	1312	59.5
High school	569	25.8
>High school	325	14.7
**Race/ethnicity**		
Black African	989	44.8
Colored/mixed ancestry	615	27.9
Indian	249	11.3
White	353	16.0
**Residence**		
Urban	1701	77.1
Rural	505	22.9
**Smoking status** (n=2203)		
Ex-smoker	264	12.0
Current smoker	1939	88.0
**Ever smoking status by year**		
2007 (n=764)		
Ex-smoker	82	10.7
Current smoker	682	89.3
2010 (n=705)		
Ex-smoker	81	11.5
Current smoker	624	88.5
2017 (n=734)		
Ex-smoker	101	13.8
Current smoker	633	86.2
**Past month exposure to others’ smoking** (n=2024)		
No exposure	240	10
Exposed at work or public places only	324	17.8
Exposed at home (with or without other places)	1460	72.2
**Ever-used e-cigarettes during 2010 & 2017** (n=1439)		
Yes	102	7.1
No	1337	92.9

* Not always 2206 because of missing data elements.

Current smoking did not significantly change during 2007 and 2017 (p=0.171). Trends in the smoked CPD, receipt of quit advice, planning to quit, quit attempts and use of e-cigarette are shown in Table 2. These show that the average smoked CPD did not also change significantly between 2007 and 2017 (Table 3). While the highest smoked CPD was consistently significantly associated with self-identifying as White across all the three survey years, higher smoked CPD was significantly associated with residing in an urban area and having obtained greater than high school education only in 2007 and 2010. These socioeconomic differences in smoked CPD were no longer statistically significant by 2017 (Table 3).

**Table 2:** Smoking behaviors, quit attempts, advice by a healthcare professional and use of e-cigarettes among current smokers in South Africa, 2007–2017

*Current smoker % (95% CI)*	*CPD Mean (SD)*	*Quit attempt % (n)*	*Quit plan % (n)*	*Advised % (n)*	*Ever used e-cigs (2010 & 2017) % (n)*
		**N=1881**			**N=1257**
**2007 (n=682)**					
20.8 (18.6–23.1)	9.18 (0.41)	60.7 (447)	55.6 (391)	26.2 (195)	-
**2010 (n=624)**					
17.9 (15.9–20.0)	9.34 (0.47)	54.5 (311)	45.7 (271)	23.2 (161)	4.3 (29)
**2017 (n=633)**					
20.1 (17.9–22.4)	8.69 (0.56)	60.0 (361)	49.8 (301)	23.7 (144)	8.4 (48)
p=0.171	0.661	0.399	0.090	0.701	**0.05**

CPD: cigarettes per day. SD: standard deviation. E-cigs: e-cigarettes.

**Table 3:** Smoking behaviors, quit attempts, advice by a healthcare professional and use of e-cigarettes among current smokers in South Africa, 2007, 2010 and 2017

*Characteristics*	*2007*				*2010*				*2017*				*2010 & 2017 (pooled data)*
	*CPD Mean (SD)*	*Quit attempt % (n)*	*Quit plan % (n)*	*Quit advice % (n)*	*CPD Mean (SD)*	*Quit attempt % (n)*	*Quit plan % (n)*	*Quit advice % (n)*	*CPD Mean (SD)*	*Quit attempt % (n)*	*Quit plan % (n)*	*Quit advice % (n)*	*Ever e-cig use % (n)*
**Age** (years)													
16–24	8.71 (0.94)	50.0 (74)	58.9 (83)	9.8 (20)	7.36 (062)	57.5 (60)	42.3 (51)	20.5 (18)	8.59 (1.14)	70.2 (67)	62.2 (64)	25.0 (23)	10.3 (19)
25–34	8.30 (0.54)	68.2 (116)	72.8 (118)	29.1 (45)	8.56 (0.68)	51.0 (71)	57.2 (73)	8.9 (19)	7.93 (0.84)	55.3 (71)	43.5 (67)	14.3 (24)	5.5 (16)
35–44	8.74 (0.63)	86.3 (106)	60.8 (98)	27.6 (55)	10.48 (1.12)	61.1 (71)	54.6 (65)	25.4 (35)	10.52 (1.74)	56.6 (70)	57.2 (72)	16.4 (28)	4.6 (15)
45–54	9.89 (0.97)	46.1 (64)	36.6 (59)	37.4 (45)	10.11 (0.92)	48.2 (43)	36.1 (37)	35.4 (37)	8.19 (0.70)	58.0 (66)	43.9 (60)	31.9 (48)	5.8 (14)
≥55	11.59 (1.21)	69.9 (87)	50.8 (70)	31.6 (60)	11.61 (1.04)	51.6 (43)	31.3 (48)	37.5 (54)	8.16 (0.70)	58.7 (87)	43.3 (75)	35.4 (44)	5.9 (13)
p	0.14	**0.016**	**0.001**	**0.003**	**0.002**	0.616	**0.022**	**0.002**	0.661	0.446	0.150	**0.029**	0.301
**Residence**													
Rural	6.60 (0.45)	63.0 (117)	56.4 (119)	21.3 (56)	7.82 (0.78)	55.4 (61)	40.5 (56)	24.2 (26)	7.33 (0.92)	68.6 (253)	54.1 (60)	27.0 (28)	5.4 (14)
Urban	10.16 (0.53)	59.7 (330)	58.5 (209)	28.7 (169)	9.85 (0.55)	54.2 (250)	47.8 (218)	23.2 (137)	9.086 (0.66)	46.9 (108)	49.2 (278)	80.7 (131)	7.0 (63)
p	**<0.001**	0.562	0.716	0.110	**0.034**	0.854	0.288	0.869	0.124	0.209	0.500	0.345	0.536
**Gender**													
Male	8.92 (0.47)	60.7 (282)	54.8 (269)	23.3 (124)	9.33 (0.50)	55.5 (190)	44.5 (160)	21.7 (190)	8.90 (0.66)	63.6 (253)	50.2 (212)	23.6 (104)	6.6 (51)
Female	10.60 (0.71)	60.7 (165)	67.1 (159)	36.1 (101)	9.4 (0.79)	52.1 (121)	49.5 (114)	27.8 (73)	7.69 (0.64)	46.9 (108)	51.5 (120)	18.1 (55)	6.6 (26)
p	0.204	0.561	0.056	**0.019**	0.930	0.589	0.442	0.234	0.178	**0.033**	0.851	0.291	0.987
**Race**													
Black African	7.36 (0.40)	61.8 (203)	60.4 (215)	26.4 (90)	7.54 (0.49)	55.7 (121)	47.2 (110)	22.6 (49)	7.05 (0.40)	67.0 (175)	59.0 (165)	24.6 (71)	5.7 (32)
Colored	9.31 (0.73)	60.1 (135)	64.4 (124)	24.2 (58)	8.32 (0.64)	52.6 (97)	46.3 (90)	26.2 (23)	8.83 (1.10)	35.0 (80)	37.4 (85)	16.3 (39)	2.4 (11)
Indian/Asian	9.53 (0.75)	67.2 (51)	61.6 (47)	49.6 (46)	8.93 (0.50)	61.7 (37)	62.0 (34)	16.4 (23)	8.67 (1.28)	77.4 (48)	75.7 (47)	39.9 (28)	18.3 (13)
White	16.60 (0.95)	55.0 (58)	37.3 (42)	24.0 (31)	16.50 (1.04)	51.0 (56)	38.0 (40)	24.6 (33)	14.67 (2.46)	56.3 (58)	25.4 (41)	16.0 (21)	11.9 (21)
p	**<0.001**	0.690	**0.012**	0.140	**<0.001**	0.749	0.300	0.732	**0.005**	**<0.001**	**<0.001**	0.204	**0.002**
**Education**													
<High school	8.45 (0.47)	60.7 (295)	58.8 (288)	26.1 (152)	8.07 (0.55)	52.3 (161)	44.41 (140)	27.3 (96)	8.17 (0.60)	60.7 (206)	66.6 (36)	27.7 (97)	6.7 (33)
High school	9.96 (0.84)	60.6 (104)	54.1 (95)	24.0 (43)	10.73 (0.9)	58.2 (94)	44.0 (86)	15.4 (34)	8.08 (0.63)	54.2 (100)	60.7 (51)	11.9 (37)	5.1 (23)
>High school	11.44 (1.23)	61.0 (48)	62.7 (45)	33.6 (30)	10.94 (0.91)	55.6 (56)	53.7 (48)	24.5 (323)	12.51 (2.67)	77.7 (55)	46.3 (245)	22.9 (25)	9.1 (21)
p	**0.011**	0.999	0.659	0.503	**0.004**	0.664	0.454	0.10	0.256	0.197	0.543	**0.008**	0.382
**Ever e-cig use**													
No	-	-	-	-	9.31 (0.47)	54.6 (295)	45.6 (28)	23.0 (154)	8.56 (0.54)	58.7 (334)	50.6 (314)	21.5 (147)	-
Yes	-	-	-	-	10.25 (1.79)	53.4 (15)	54.9 (16)	31.8 (8)	10.10 (1.76)	85.6 (25)	45.3 (20)	33.5 (12)	-
p					0.612	0.923	0.443	0.466	0.361	**0.002**	0.668	0.269	-
**Advised to quit**													
No	9.02 (0.47)	56.1 (27)	54.5 (271)	-	9.38 (0.52)	51.0 (220)	44.4 (196)	-	8.53 (0.52)	55.2 (240)	45.7 (214)	-	4.3 (43)
Yes	9.59 (0.73)	76.6 (158)	65.8 (142)	-	9.23 (0.80)	65.8 (91)	50.8 (78)	-	9.49 (1.56)	73.6 (104)	61.7 (101)	-	9.8 (63)
p	0.509	**0.001**	0.083	-	0.869	**0.012**	0.340	-	0.551	**0.026**	**0.033**	-	**0.033**
**Exposure to others’ smoking**													
No exposure	9.28 (1.60)	70.0 (52)	83.0 (50)	32.8 (27)	11.61 (1.67)	46.6 (19)	22.7 (10)	11.2 (6)	11.67 (3.84)	74.4 (29)	66.6 (36)	16.4 (17)	15.1 (15)
Exposed at work or public places only	8.46 (0.68)	70.8 (73)	65.6 (69)	22.3 (27)	9.06 (1.05)	62.1 (47)	53.9 (46)	35.7 (25)	6.70 (0.71)	79.8 (49)	60.7 (51)	32.3 (23)	11.6 (15)
Exposed at home (with or without other exposures)	9.48 (0.59)	58.9 (261)	52.8 (248)	28.7 (136)	9.34 (0.53)	53.9 (222)	44.1 (195)	22.9 (120)	8.67 (0.50	55.3 (279)	46.3 (245)	21.0 (116)	4.9 (45)
p	0.630	0.236	**0.012**	0.489	0.392	0.447	0.084	0.057	0.062	**0.001**	0.059	0.215	**0.009**

E-cig: e-cigarette. Significant p (p<0.05) in bold.

On aggregate, there was no significant change in the proportion of current smokers who received quit advice from a healthcare professional between 2007 and 2017. Across all three survey years, the proportion of smokers who reported to have received quit advice was consistently highest among those aged ≥45 years (Table 3). A significantly greater proportion of females received quit advice during 2007, but no significant gender differences were observed in more recent years. During 2017, a significantly greater portion of those with less than high school education and ever e-cigarette users reported to have received quit advice.

The proportion of smokers planning or making quit attempts did not change significantly between 2007 and 2017. The lowest proportion of those planning to quit smoking were aged 45–54 years for 2007 and 2010, but no significant differences in age was detected in the proportion of smokers planning to quit smoking in 2017. Similarly, the lowest proportion of those planning to quit were among those who self-identified as White during 2007 and 2017, but this proportion was not significantly lower than others during 2010. During 2017, the lowest proportions of smokers who attempted to quit were among those who reported not to have ever used e-cigarettes, females, those who self-identified as Coloreds and reported exposure to others’ smoking at home. None of these group differences was statistically significant in earlier survey years. However, consistent across all survey years is that a significantly higher proportion of those who reported to have received quit advice had made a quit attempt (Table 3).

The prevalence of ever use of e-cigarette among smokers was marginally significantly lower in 2010 than in 2017 (4.3% vs 8.4%; p=0.05). On aggregate, ever e-cigarette use was significantly higher among smokers who self-identified as Indian/Asian or White than among those who self-identified as Colored or Black African. Similarly, ever e-cigarette use was significantly higher among smokers who reported to have received quit advice than among those that had not (4.3 vs 9.8%; p=0.03).

In the final multivariable logistic regression, controlling for survey year (Table 4), reporting having received brief advice (OR=1.967; 95% CI: 1.255–3.083), and being of Colored/mixed race/ethnicity (OR=0.467: 95% CI: 0.298–0.732) compared to being Black African, were independently associated with making a quit-attempt.

**Table 4:** Final multivariable-adjusted logistic regression models with quit attempt as outcome

*Variable*	*OR (95% CI)*
**Survey year**	
2010	1
2016	0.813 (0.551–1.199)
**Race/ethnicity**	
Black African	1
Colored/mixed ancestry	**0.467 (0.298–0.732)**
Indian/Asian	1.336 (0.735–2.429)
White	0.705 (0.445–1.117)
**Ever e-cig use**	
No	
Yes	1.924 (0.977–3.789)
**Quit advice**	
No	1
Yes	**1.967 (1.255–3.083)**

E-cig: e-cigarette. Significant p (p<0.05) in bold.

## DISCUSSION

This study found that there has been no significant change in South Africa in the proportion of adult smokers offered quit advice, those planning to quit and those making quit attempts since the adoption of the WHO FCTC Article 14 guidelines in 2010. Furthermore, ever use of e-cigarettes significantly increased but was not independently associated with significantly higher odds of making a quit attempt. Considering that quit advice provided by a healthcare professional promotes a quit attempt and increases the odds of a successful attempt^9^, these findings show the failure of the South African health system to harness the clinical, public health and economic benefits of this smoking cessation intervention for improved personal and country level health outcomes during the study period. There is, therefore, a dire need for strategies to scale up the offer of quit advice in health facilities, especially since <25% of smokers were offered quit advice during the period under review. However, to be successful, such strategies must address healthcare professionals’ personal limitations (e.g. poor knowledge and skills), health systems constraints (e.g. patient overload, lack of time and treatment resources), sociocultural barriers, misconceptions and misperceptions about the effectiveness of tobacco dependence treatments^22^.

The findings of this study also suggest that healthcare professionals may be selective in whom they advise. First, older smokers may be perceived to be at higher risk of ill-health and therefore prioritized for advice. Second, although people with higher educational attainments are generally more likely to engage their healthcare professionals on health issues^23^, smokers with the lowest educational attainment in South Africa may be more pressured to quit because of the more severe negative financial effects of tax and price increases resulting from the tobacco control policy related to excise taxes. Third, smokers who may be those contemplating quitting may engage their healthcare professional more intensely, resulting in being advised to quit but might use e-cigarettes to manage nicotine withdrawal as they make quit attempts^13^. While health system constraints have been reported to make selective advice convenient for healthcare professionals, it can increase missed opportunities since those smokers perceived not to fit preconceived profiles may not be advised. Therefore, as envisaged in the 2010 WHO FCTC Article 14 guidelines, all smokers need to be advised to quit at every opportunity, especially since most smokers need several quit attempts to successfully quit.

In this study, the trends in quit attempts across sociodemographic characteristics may be explained in several ways. It was interesting to note that while in 2007, the lowest proportion of those making quit attempts was among those aged 16–24 years and 45–54 years, by 2017 there were as many making quit attempts in these age groups as in the other age groups. Considering that reporting having received advice to quit was consistently highest among those ≥45 years, the fact that those aged 45–54 years in recent years had equally begun to make quit attempts may suggest an increasing health-risk awareness among these middle-aged adults who are more likely to have started developing chronic health conditions from many years of smoking. Similarly, just as many amongst those aged 16–24 years are now making quit attempts, which might suggest that they are beginning to heed the call to avoid becoming addicted and thus quit smoking before adverse health outcomes set in. It might also be related to increasing promotion of modified risk tobacco products (MRTP) to these young adults as reduced-harm alternatives to cigarette smoking. This is particularly so, because this recent increase in the proportion of younger adults making a quit attempt coincided with the period when these products were beginning to gain popularity in the South African market. There is, however, need for more studies to ascertain the relationship between increasing availability of these MRTPs and relatively increasing quit attempts among younger South African adults, especially that e-cigarette was not significantly associated with quit attempt in a controlled analysis. Also, subsections of the South African population that are known to have high smoking prevalence such as Colored/mixed race or White^7^, may have higher nicotine dependence and difficulties in coping with withdrawal symptoms, and therefore make fewer quit attempts. This may be compounded by the high possibility of dual use of cigarettes and other substance use (e.g. alcohol)^24^, which makes nicotine dependence more severe, promoting continued smoking and fewer quit attempts in this population group^25^.

The CPD patterns in this study highlight a few issues – they confirm previous analyses that most smokers in South Africa are ‘light smokers’ and may therefore be motivated to quit, if offered brief advice^26^. Also the association between higher number of smoked CPD and higher socioeconomic status (SES), reflected in this study as having attained greater than high school education and living in urban areas, may suggest higher level of dependence among those of higher SES, which is consistent with what was recently reported in South Africa^26^. However, others have also reported the contrary, i.e. a higher level of nicotine dependence among those of lower SES^27^. Therefore, our observed trend during 2007 and 2010, may just be a reflection of affordability of more cigarettes by those of higher SES. This hypothesis would be consistent with the fact that white South Africans who are the most economically well-off racial group^28^, have consistently reported the highest amount of smoked CPD. The lack of significant difference in smoked CPD between lower and higher SES in 2017 may reflect increasing relative affordability since there has been no significant increase in excise taxes and thus in the real price of cigarettes over the last decade in South Africa^29^. An alternative hypothesis is that those of lower SES (as represented by those with lower than high school education) may be smoking as many CPD as higher SES smokers in 2017 because of increasing nicotine dependence, since they were no more likely to have made a quit attempt despite being most frequently advised to quit.

The significantly higher uptake of e-cigarettes among Indians/Asians in this study (Table 3) may reflect a search for an alternative and less harmful behavior to smoking, possibly underscored by the self-awareness of a higher cardiovascular risk among South African Indians/Asians compared to other ethnic groups^30^. Ever use of e-cigarettes in this study was most prevalent among young smokers aged 16–24 years, most of whom were also smoking cigarettes at frequency no less than never users of e-cigarettes. Without the benefit of their smoking history before ever e-cigarette use, it may well be that ever use of e-cigarettes might have reduced the number of cigarettes smoked by these young smokers. Although the order of uptake behavior is not known in this study, others have suggested that young people often begin to use e-cigarettes before cigarette smoking, thus e-cigarette use may provide a gateway to regular smoking^31,32^. Alternatively, e-cigarette use may be delaying combustible cigarette use in young people who were going to start. However, because we do not know to what extent this applies in South Africa, further studies are needed to provide an understanding of whether e-cigarette use provides a gateway to regular smoking or is used mainly to mitigate the unpleasant effects of withdrawal symptoms during quit attempts, or merely used concurrently with cigarette smoking even when quitting is not contemplated. While reporting receipt of quit advice from a health professional was more among ever users of e-cigarettes and may suggest that healthcare professionals may be directly or indirectly promoting e-cigarette use, the number of e-cigarette users in this study is small and larger study samples of e-cigarette users are needed to validate this hypothesis.

### Limitations

This study has some limitations. The study relied on self-reports that may result in information and social desirability biases, and thus affect outcome estimates. Missing data in some of the measures could have resulted in respondent bias, but sensitivity analysis did not show significant differences in the sociodemographic characteristics of those who had complete data and those who did not (data not displayed). It is possible that some who smoke other forms of non-traditional cigarettes might have not been identified as smokers and not considered for quit advice by healthcare practitioners, affecting some of the study outcome estimates. Furthermore, the small sample of current e-cigarette users precludes any meaningful conclusion regarding the associations found, more so that only ever users of e-cigarettes (that could have included those who have vaped once or few times and quit) were studied and these findings may not represent the true behaviors of current e-cigarette users. Since this study is cross-sectional, it is not known whether the co-use of e-cigarettes has led to any reductions in combustible cigarette smoking rates. Notwithstanding the limitations regarding findings on e-cigarette use, this study is based on a large and nationally representative sample and for the first time provides insights into trends in quit advice by healthcare professionals and smokers’ quit attempts in South Africa, before and after the adoption of Article 14.

## CONCLUSIONS

The adoption of the WHO FCTC Article 14 has had no significant effect on the offer of quit advice by healthcare professionals and smokers’ quit attempts in South Africa. There is a need to scale up quit advice as part of evidence-based treatment guidelines for tobacco dependence treatment in South Africa.
